# Ethnic differences in prevalence of actionable HbA1c levels in UK Biobank: implications for screening

**DOI:** 10.1136/bmjdrc-2021-002176

**Published:** 2021-08-05

**Authors:** Jana J Anderson, Paul Welsh, Frederick K Ho, Lyn D Ferguson, Claire E Welsh, Pierpaolo Pellicori, John G F Cleland, John Forbes, Stamatina Iliodromiti, James Boyle, Robert Lindsay, Carlos Celis-Morales, Stuart Robert Gray, Srinivasa Vittal Katikireddi, Jason Martin Regnald Gill, Jill P Pell, Naveed Sattar

**Affiliations:** 1 Institute of Health and Wellbeing, University of Glasgow, Glasgow, UK; 2 Institute of Cardiovascular and Medical Sciences, University of Glasgow, Glasgow, UK; 3 Population and Health Sciences, Newcastle University, Newcastle upon Tyne, UK; 4 Faculty of Education and Health Sciences, University of Limerick, Limerick, Ireland; 5 Centre of Women’s Health, Yvonne Carter Building, Queen Mary University of London, London, UK

**Keywords:** diabetes mellitus, type 2, early diagnosis, glycated hemoglobin A, ethnic groups

## Abstract

**Introduction:**

Early detection and treatment of diabetes as well as its prevention help lessen longer-term complications. We determined the prevalence of pre-diabetes and undiagnosed diabetes in the UK Biobank and standardized the results to the UK general population.

**Research design and methods:**

This cross-sectional study analyzed baseline UK Biobank data on plasma glycated hemoglobin (HbA1c) to compare the prevalence of pre-diabetes and undiagnosed diabetes mellitus in white, South Asian, black, and Chinese participants. The overall and ethnic-specific results were standardized to the UK general population aged 40–70 years of age.

**Results:**

Within the UK Biobank, the overall crude prevalence was 3.6% for pre-diabetes, 0.8% for undiagnosed diabetes, and 4.4% for either. Following standardization to the UK general population, the results were similar at 3.8%, 0.8%, and 4.7%, respectively. Crude prevalence was much higher in South Asian (11.0% pre-diabetes; 3.6% undiagnosed diabetes; 14.6% either) or black (13.8% pre-diabetes; 3.0% undiagnosed diabetes; 16.8% either) participants. Only six middle-aged or old-aged South Asian individuals or seven black would need to be tested to identify an HbA1c result that merits action.

**Conclusions:**

Single-stage population screening for pre-diabetes or undiagnosed diabetes in middle-old or old-aged South Asian and black individuals using HbA1c could be efficient and should be considered.

**Significance of this study**

**What is already known about this subject?**

- Diabetes prevention programs now operate in many countries, including the UK.
- How to efficiently screen for people at elevated risk and in different ethnicities remains uncertain.

**What are the new findings?**

- Overall, we estimate 1 in 22 (4.7%) of individuals aged 40–70 years old in the UK have actionable glycated hemoglobin (HbA1c) concentrations.
- This prevalence is markedly higher in minority ethnic groups.
- 1 in 6–7 individuals of black or South Asian ethnicity have actionable values and approximately 1 in 30 are living with undiagnosed diabetes.

**How might these results change the focus of research or clinical practice?**

- HbA1c could be used to identify pre-diabetes and undiagnosed diabetes in middle-old and old-aged South Asian and black individuals without the need for prior risk scoring.
- Similar work is now needed in other countries.

## Introduction

The prevalence of diabetes is rising worldwide, particularly in low-income and middle-income countries.^1^ Diabetes has long been recognized as a major cause of morbidity and mortality. Most recently, diabetes has been identified as a risk factor for adverse COVID-19 outcomes, including hospitalization and death.^2 3^ In 2017, it was estimated that the global prevalence of pre-diabetes was 7.7% and 4.2% for undiagnosed diabetes.^4^ It is predicted that these numbers will rise significantly by 2045.^4^

There is now clear evidence that people with pre-diabetes are not just at elevated risk of developing diabetes but also at risk of adverse cardiovascular outcomes.^5^ Lifestyle changes and metformin can prevent progression to diabetes,^6^ and diabetes prevention programs now operate in many countries, including the UK.^7^ However, the scale of pre-diabetes and undiagnosed diabetes and the best way to identify people with these conditions are currently unclear.

The aim of the current study was to determine the prevalence of pre-diabetes and undiagnosed diabetes by ethnic group and the average number of people who would need to undergo testing to identify each case.

## Research design and methods

This study used data from the UK Biobank (https://www.ukbiobank.ac.uk/). The UK Biobank is a large general population cohort of 502 624 middle-aged or older-aged participants (40–69 years old at recruitment) who were recruited at 22 centers across the UK between March 2006 and December 2010.^8^ During recruitment, participants provided detailed demographic, lifestyle and medical history information and biological samples, and anthropometric measurements were taken. Inclusion in this study was restricted to participants who identified themselves as white, Indian, Pakistani, Bangladeshi, black African, black Caribbean, or Chinese. To provide sufficient statistical power, Indian, Pakistani, and Bangladeshi participants were amalgamated into South Asian, and black African and black Caribbean participants were amalgamated into black.

All participants gave written informed consent before enrollment in the study.

Deprivation status was based on the Townsend Deprivation Index derived from the postcode of residence at recruitment. Body mass index (BMI) was calculated from weight/height^2^ and categorized into underweight (<18.5 kg/m^2^), normal weight (18.5–24.9 kg/m^2^), overweight (25.0–29.9 kg/m^2^), and obese (≥30.0 kg/m^2^), according to the WHO classification. A lifestyle score, previously shown to be associated with all-cause mortality, was used to characterize overall lifestyle as described previously.^9 10^ Lifestyle factors, self-reported at baseline, included smoking status (current, former, or never), physical activity (time spent doing moderate and vigorous physical activity per week, converted to metabolic equivalents (METs)-min/week and dichotomized into inactive (<600 METs-min/week) and active (≥600 METs-min/week) according to the physical activity guidelines^11^), sedentary time (watching television, using a computer, and non-work-related driving), adequate sleep, and optimal dietary intake of fruit and vegetables (≥5/day), red meat (<70 g/day) and processed meat (never or less than once a week), oily fish (≥1/week), and alcohol (<14 units/week). The overall lifestyle score ranged from 0 (most unhealthy; highest risk of all-cause mortality) to 9 (most healthy; lowest risk of all-cause mortality). Sampling procedures for UK Biobank biomarkers have been described and validated previously.^12 13^ Briefly, biochemistry analyses were performed at a dedicated central laboratory on 480 000 samples between 2014 and 2017 and included plasma glycated hemoglobin (HbA1c) (VARIANT II TURBO Hemoglobin Testing System; Bio-Rad). Data were adjusted for preanalytical variables by the UK Biobank centrally before release.

Known diabetes was defined as at least one of the following: type 1 or type 2 diabetes self-reported by the participant at baseline and/or taking insulin or other diabetes-related medications (metformin, sulfonylurea, meglitinides/glinides/prandial glucose regulators, alpha-glucosidase inhibitors, thiazolidinedione/glitazones, dipeptidyl peptidase-4 (DPP-4) inhibitors/gliptins, incretin mimetics/glucagon-like peptide-1 (GLP-1) analogs, and amylin analogs). Participants with known diabetes were excluded from the study. Among the remaining participants, pre-diabetes and undiagnosed diabetes were ascertained from HbA1c concentrations and defined as HbA1c 42–47.9 mmol/mol and ≥48 mmol/mol, respectively.^14^

Sensitivity analyses were conducted excluding participants with self-reported cardiovascular disease at baseline (heart attack, angina, stroke, or transient ischemic attack), since such individuals are likely to be already in receipt of preventive pharmaceutical or lifestyle interventions.

Participants were stratified by ethnicity and sex and their baseline characteristics summarized using percentages for categorical variables and mean and SD, or median and IQR, for continuous variables. Crude prevalence was calculated by dividing the total number of cases by the total number of the population in each category. Crude prevalence was derived overall and by age, gender, and ethnic subgroup. The 2011 Census data^15^ were used to standardize the prevalence rates to the age, gender, and ethnic breakdown of the UK general population, within the age group recruited to the UK Biobank. Briefly, sex-specifc and ethnic-specific prevalence rates obtained from the UK Biobank were used to estimate the total cases in the UK general population, according to the total of men and women in each of the ethnic group in the 40–70 age group only, and the corresponding UK population prevalence rates for all 40–70 years old were recalculated. The yield (average number needed to test to detect a case) was derived from inversion of the prevalence.

## Results

UK Biobank participants who had missing data on HbA1c (n=36 135), had known diabetes at baseline (n=24 593), or classified themselves as mixed or other ethnic group (n=8081) were excluded from the study. The resultant study population comprised 433 856 participants: 419 512 (96.7%) white, 7400 (1.7%) South Asian, 5578 (1.3%) black, and 1366 (0.3%) Chinese. This compared with 94.0%, 3.2%, 2.4%, and 0.5%, respectively, in the UK general population within the same age range. White participants were older, black participants were more likely to live in the most deprived areas, and black women had the highest BMI (table 1). Lifestyle score was higher (healthier) among women than men but did not differ significantly by ethnic group.

**Table 1:** Characteristics of UK Biobank participants by ethnic group and sex, excluding people with known diabetes at baseline

	Women				Men			
	White	South Asian	Black	Chinese	White	South Asian	Black	Chinese
	n=231 137	n=3559	n=3210	n=868	n=188 375	n=3841	n=2368	n=498
Age, years, mean (SD)	56.5 (8.0)	52.8 (8.1)	51.6 (7.7)	52.3 (7.4)	56.7 (8.2)	52.4 (8.5)	51.1 (7.9)	51.9 (7.9)
BMI, kg/m^2^, mean (SD)	27.0 (5.0)	27.0 (4.7)	30.1 (5.8)	23.3 (3.4)	27.6 (4.1)	26.7 (3.7)	28.3 (4.2)	25.1 (3.0)
Deprivation quintile, n (%)								
1 (least deprived)	48 496 (21.0)	370 (10.4)	92 (2.9)	129 (14.9)	40 129 (21.3)	349 (9.1)	75 (3.2)	74 (14.9)
2	48 054 (20.8)	367 (10.3)	155 (4.8)	134 (15.5)	39 211 (20.8)	368 (9.6)	111 (4.7)	86 (17.3)
3	47 978 (20.8)	547 (15.4)	263 (8.2)	145 (16.8)	38 128 (20.3)	539 (14.1)	190 (8.1)	67 (13.5)
4	46 105 (20.0)	1053 (29.6)	698 (21.8)	221 (25.6)	36 477 (19.4)	1062 (27.7)	508 (21.5)	126 (25.4)
5 (most deprived)	40 239 (17.4)	1217 (34.2)	1999 (62.3)	236 (27.3)	34 195 (18.2)	1515 (39.5)	1477 (62.6)	143 (28.8)
Lifestyle score, n (%)								
0–1 (least healthy)	42 (0.02)	1 (0.03)	1 (0.03)	0.0 (0.00)	275 (0.2)	2 (0.1)	8 (0.4)	2 (0.4)
2–3	2844 (1.3)	15 (0.5)	54 (1.8)	5 (0.6)	8630 (4.7)	108 (3.1)	129 (6.1)	10 (2.1)
4–5	34 167 (15.0)	344 (10.4)	556 (18.5)	99 (12.0)	54 786 (29.5)	766 (22.0)	676 (31.7)	111 (23.3)
6–7	117 573 (51.5)	1915 (57.7)	1449 (48.3)	432 (52.2)	94 069 (50.7)	1866 (53.6)	975 (45.7)	242 (50.8)
8–9 (most healthy)	73 627 (32.3)	1046 (32.3)	939 (31.3)	291 (35.2)	27 699 (15.0)	741 (21.3)	344 (16.1)	111 (23.3)
HbA1c, median (IQR)	35.0 (32.6–37.3)	37.1 (34.3–40.0)	37.1 (34.0–40.4)	36.2 (33.6–38.9)	34.9 (32.6–37.3)	37.2 (34.5–40.0)	37.6 (34.3–40.6)	36.8 (34.2–39.2)
HbA1c category, n (%), WHO criteria								
Normal (<42)	222 806 (96.4)	3060 (86.0)	2668 (83.1)	796 (91.7)	179 961 (95.5)	3262 (84.9)	1973 (83.3)	449 (90.2)
Pre-diabetes (42–47.9)	7227 (3.1)	383 (10.8)	452 (14.1)	61 (7.0)	6617 (3.5)	432 (11.3)	318 (13.4)	45 (9.0)
Undiagnosed diabetes (≥48)	1104 (0.5)	116 (3.3)	90 (2.8)	11 (1.3)	1797 (1.0)	147 (3.8)	77 (3.3)	4 (0.8)
HbA1c category, n (%), ADA criteria								
Normal (<39)	199 990 (86.5)	2391 (67.2)	2076 (64.7)	652 (75.1)	161 751 (85.9)	2548 (66.3)	1446 (61.1)	358 (71.9)
Pre-diabetes (39–47.9)	30.043 (13.0)	1.052 (29.6)	1044 (35.5)	205 (23.6)	24 827 (13.2)	1146 (29.8)	845 (35.7)	136 (27.3)
Undiagnosed diabetes (≥48)	1104 (0.5)	116 (3.3)	90 (2.8)	11 (1.3)	1797 (1.0)	147 (3.8)	77 (3.3)	4 (0.8)

HbA1c measured in mmol/L.

ADA, American Diabetes Association; BMI, body mass index; HbA1c, glycated hemoglobin; n, number.

Overall, the crude prevalence was 3.6% for pre-diabetes, 0.8% for undiagnosed diabetes, and 4.4% for either (table 2). However, there were wide variations by ethnic group. The prevalence of pre-diabetes was only 3.3% among white participants compared with 11.0% and 13.8% in South Asian and black, respectively. Similarly, only 0.7% of white participants had undiagnosed diabetes compared with 3.6% of South Asian and 3.0% of black participants. As a result, 25 white participants would need to be tested to identify one case of either pre-diabetes or undiagnosed diabetes compared with only 7 South Asian participants or 6 black participants. Standardization against the UK general population in the same age range made little difference to the overall or ethnic-specific results (table 3).

**Table 2:** Crude prevalence of pre-diabetes and undiagnosed diabetes and crude yield of HbA1c in the UK Biobank by ethnic group

	White n=419 512	South Asian n=7400	Black n=5578	Chinese n=1366	Overall n=433 856
	Cases, n Prevalence (95% CI)	Cases, n Prevalence (95% CI)	Cases, n Prevalence (95% CI)	Cases, n Prevalence (95% CI)	Cases, n Prevalence (95% CI)
Pre-diabetes	13 844 3.3 (3.3 to 3.4)	815 11.0 (10.3 to 11.8)	770 13.8 (12.9 to 14.7)	106 7.8 (6.5 to 9.3)	15 535 3.6 (3.5 to 3.6)
Undiagnosed diabetes	2901 0.7 (0.7 to 0.7)	263 3.6 (3.2 to 4.0)	167 3.0 (2.6 to 3.5)	15 1.1 (0.7 to 1.8)	3346 0.8 (0.8 to 0.8)
Pre-diabetes or undiagnosed diabetes	16 745 4.0 (3.9 to 4.1)	1078 14.6 (13.5 to 15.8)	937 16.8 (15.5 to 18.2)	121 8.9 (7.1 to 11.1)	18 881 4.4 (4.3 to 4.4)
	**Yield**	**Yield**	**Yield**	**Yield**	**Yield**
Pre-diabetes	1 in 30	1 in 9	1 in 7	1 in 13	1 in 28
Undiagnosed diabetes	1 in 143	1 in 28	1 in 33	1 in 91	1 in 130
Pre-diabetes or undiagnosed diabetes	1 in 25	1 in 7	1 in 6	1 in 11	1 in 23

Yield refers to the average number needing to be tested to identify one case.

HbA1c, glycated hemoglobin; n, number.

**Table 3:** Prevalence of pre-diabetes and undiagnosed diabetes and yield of HbA1c by ethnic group standardized* to the UK general population aged 40–70 years

	White n=24 119 971	South Asian n=815 891	Black n=602 358	Chinese n=117 430	Overall n=25 655 650	Overall n=25 655 650
	n (%)	n (%)	n (%)	n (%)	n (%)	Yield
Pre-diabetes	795 965 (3.3)	89 859 (11.0)	83 151 (13.8)	9112 (7.8)	978 087 (3.8)	1 in 26
Undiagnosed diabetes	166 794 (0.7)	28 997 (3.6)	18 034 (3.0)	1290 (1.1)	215 115 (0.8)	1 in 125
Pre-diabetes or undiagnosed diabetes	962 759 (4.0)	118 856 (14.6)	101 185 (16.8)	10 402 (8.9)	1 193 202 (4.7)	1 in 22

Yield refers to the average number needing to be tested to identify one case.

*Ethnic-specific results standardized by age and sex; overall results standardized by age, sex, and ethnic group.

HbA1c, glycated hemoglobin; n, number.

When the results were recalculated for age and sex subgroups within each ethnicity group, there was a consistent trend in white and South Asian participants, whereby the prevalence of pre-diabetes or undiagnosed diabetes was higher in men than in women and increased with increasing age (tables 4 and 5). Among South Asian or black men over 55 years of age, only three to five individuals needed to be tested to identify an actionable HbA1c value.

**Table 4:** Crude prevalence of pre-diabetes and undiagnosed diabetes and crude yield of HbA1c in the UK Biobank in women by age and ethnic group

	White (n by age group)	South Asian (n by age group)	Black (n by age group)	Chinese (n by age group)	Overall (n by age group)
	22 737/66 796/100 352/41 252	n=705/1388/1107/359	n=668/1513/761/268	n=159/357/291/61	n=24 269/70 054/102 511/41 940
	n (%)	n (%)	n (%)	n (%)	n (%)
Pre-diabetes					
Age (years)					
40–44	118 (0.5)	28 (4.0)	33 (4.9)	2 (1.3)	181 (0.7)
45–54	1001 (1.5)	97 (7.0)	185 (12.2)	9 (2.5)	1292 (1.8)
55–64	3692 (3.7)	186 (16.8)	157 (20.6)	41 (14.1)	4076 (4.0)
65–74	2416 (5.9)	72 (20.1)	77 (28.7)	9 (14.8)	2574 (6.1)
Undiagnosed diabetes					
40–44	30 (0.1)	8 (1.1)	6 (0.9)	0 (0)	44 (0.2)
45–54	183 (0.3)	39 (2.8)	36 (2.4)	3 (0.8)	261 (0.4)
55–64	588 (0.6)	52 (4.7)	32 (4.2)	7 (2.4)	679 (0.7)
65–74	303 (0.7)	17 (4.7)	16 (6.0)	1 (1.6)	337 (0.8)
Pre-diabetes or undiagnosed diabetes					
40–44	148 (0.7)	36 (5.1)	39 (5.8)	2 (1.3)	225 (0.9)
45–54	1184 (1.8)	136 (9.8)	221 (14.6)	12 (3.4)	1553 (2.2)
55–64	4280 (4.3)	238 (21.5)	189 (24.8)	48 (16.5)	4755 (4.6)
65–74	2719 (6.6)	89 (24.8)	93 (34.7)	10 (16.3)	2911 (6.9)
	**Yield**	**Yield**	**Yield**	**Yield**	**Yield**
Pre-diabetes					
40–44	1 in 200	1 in 25	1 in 20	1 in 77	1 in 143
45–54	1 in 67	1 in 14	1 in 8	1 in 40	1 in 56
55–64	1 in 27	1 in 6	1 in 5	1 in 7	1 in 25
65–74	1 in 17	1 in 5	1 in 4	1 in 7	1 in 16
Undiagnosed diabetes					
40–44	1 in 1000	1 in 91	1 in 111	N/A	1 in 500
45–54	1 in 333	1 in 36	1 in 42	1 in 125	1 in 250
55–64	1 in 167	1 in 21	1 in 24	1 in 42	1 in 143
65–74	1 in 143	1 in 21	1 in 17	1 in 63	1 in 125
Pre-diabetes or undiagnosed diabetes					
40–44	1 in 143	1 in 20	1 in 17	1 in 77	1 in 111
45–54	1 in 56	1 in 10	1 in 7	1 in 30	1 in 46
55–64	1 in 23	1 in 5	1 in 4	1 in 6	1 in 22
65–74	1 in 15	1 in 4	1 in 3	1 in 6	1 in 15

Yield refers to the average number needing to be tested to identify one case.

HbA1c, glycated hemoglobin; n, number; N/A, not available.

**Table 5:** Crude prevalence of pre-diabetes and undiagnosed diabetes and crude yield of HbA1c in the UK Biobank in men by age and ethnic group

	White	South Asian	Black	Chinese	Overall
	n=19 399/51 436/79 274/38 262	n=897/1445/1042/457	n=590/1042/538/198	n=104/205/145/44	n=20 990/54 128/80 999/38 961
	n (%)	n (%)	n (%)	n (%)	n (%)
Pre-diabetes					
Age (years)					
40–44	187 (1.0)	58 (6.5)	49 (8.3)	8 (7.7)	302 (1.4)
45–54	1062 (2.1)	148 (10.2)	125 (12.0)	7 (3.4)	1342 (2.5)
55–64	3156 (4.0)	152 (14.6)	102 (19.0)	22 (15.2)	3432 (4.2)
65–74	2212 (5.8)	74 (16.2)	42 (21.2)	8 (18.2)	2336 (6.0)
Undiagnosed diabetes					
40–44	104 (0.5)	22 (2.5)	9 (1.5)	0 (0)	135 (0.6)
45–54	404 (0.8)	61 (4.2)	31 (3.0)	1 (0.5)	497 (0.9)
55–64	849 (1.1)	44 (4.2)	22 (4.1)	2 (1.4)	917 (1.1)
65–74	440 (1.1)	20 (4.4)	15 (7.8)	1 (2.3)	476 (1.2)
Pre-diabetes or undiagnosed diabetes					
40–44	291 (1.5)	80 (8.9)	58 (9.8)	8 (7.7)	437 (2.1)
45–54	1466 (2.9)	209 (14.5)	156 (15.0)	8 (3.9)	1839 (3.4)
55–64	4005 (5.1)	196 (18.8)	124 (23.1)	24 (16.6)	4349 (5.4)
65–74	2652 (6.9)	94 (20.6)	57 (28.8)	9 (20.5)	2812 (7.2)
	**Yield**	**Yield**	**Yield**	**Yield**	**Yield**
Pre-diabetes					
40–44	1 in 100	1 in 15	1 in 12	1 in 13	1 in 71
45–54	1 in 48	1 in 10	1 in 8	1 in 29	1 in 40
55–64	1 in 25	1 in 7	1 in 5	1 in 7	1 in 24
65–74	1 in 17	1 in 6	1 in 5	1 in 6	1 in 17
Undiagnosed diabetes					
40–44	1 in 200	1 in 40	1 in 67	N/A	1 in 48
45–54	1 in 125	1 in 24	1 in 33	1 in 200	1 in 29
55–64	1 in 91	1 in 24	1 in 24	1 in 71	1 in 19
65–74	1 in 91	1 in 23	1 in 13	1 in 44	1 in 14
Pre-diabetes or undiagnosed diabetes					
40–44	1 in 67	1 in 11	1 in 10	1 in 13	1 in 48
45–54	1 in 35	1 in 7	1 in 7	1 in 26	1 in 29
55–64	1 in 20	1 in 5	1 in 4	1 in 5	1 in 19
65–74	1 in 15	1 in 5	1 in 4	1 in 6	1 in 14

Yield refers to the average number needing to be tested to identify one case.

HbA1c, glycated hemoglobin; n, number; N/A, not available.

The sensitivity analyses, which excluded 22 873 participants with self-reported cardiovascular disease at baseline, made little meaningful difference to the prevalence rates or the average number needed to be tested to identify cases both overall and by ethnic group (online supplemental table S1).

10.1136/bmjdrc-2021-002176.supp1 Supplementary data

To investigate the impact of the characteristics that differed among the included ethnic groups on their increased risk of pre-diabetes and undiagnosed diabetes, we ran logistic regression models unadjusted and adjusted for sociodemographic variables (sex, age, deprivation) and lifestyle (lifestyle score). All ethnic groups had a much stronger risk of pre-diabetes or undiagnosed diabetes than white participants. After adjustment, the risk of both pre-diabetes and undiagnosed diabetes became even stronger for all ethnic groups but black participants, where it attenuated slightly (online supplemental table S2).

## Conclusions

Our findings suggest that 1 in 22 (4.7%) of individuals aged 40–70 years old in the UK have actionable HbA1c concentrations. More importantly, 1 in 6–7 individuals of black or South Asian ethnicity have actionable values and approximately 1 in 30 are living with undiagnosed diabetes. The risk of having pre-diabetes or undiagnosed diabetes is magnified by older age, male sex, living in deprived areas, and having unhealthy lifestyle. Given that diabetes prevention programs are now increasingly well developed, including the English program,^7^ these data suggest that screening based on ethnicity and age would be extremely efficient at identifying people at high risk of developing type 2 diabetes. For white individuals, needing to test 25 individuals to detect an actionable HbA1c value suggests a two-stage process may be more cost-effective, in line with the current National Institute for Health and Care Excellence guidelines,^16^ with the first stage being a simple risk score (which could easily be self-completed). Such a score would identify those at higher risk of prevalent diabetes and therefore at higher risk of being in the pre-diabetes stage since there is a continuum of risk.

The benefit of detecting those at higher risk of diabetes to delay or prevent conversion to frank diabetes has been widely accepted. Follow-up of earlier prevention studies has now shown that diabetes prevention lessens cardiovascular outcomes and lowers mortality risk.^17^ While most such studies used oral glucose tolerance test-based criteria to determine those at elevated risk of diabetes, higher HbA1c levels are well accepted in predicting incident cardiovascular disease^18^ and microvascular damage,^19^ at least as well as other glycemia measures. As HbA1c can be done any time of the day, irrespective of fasting status, it is a more feasible test for widespread community-based adoption.

Of course, HbA1c is more expensive than blood glucose and so its use for screening purposes should be weighed carefully against the cost of mass testing. That noted, given that one in six individuals of black ethnicity or one in seven of South Asian ethnicity in the 40–70 year-old age ranges have actionable HbA1c levels, it would potentially make widespread HbA1c testing in these populations cost-effective, and especially if in addition to preventing some progressing to diabetes, it is also possible to identify and treat those with undiagnosed diabetes. Many people are still not diagnosed until the development of serious complications. The importance of identifying people with undiagnosed diabetes is reinforced by the emergence of evidence-based approaches to diabetes remission and earlier intervention with pharmacological therapies that reduce the risk of progression of complications. That UK Biobank does not cover people older than 70 years of age may be seen as a limitation; however, it is notable that those with younger-onset type 2 diabetes lose more life years from diabetes than those who develop it when much older.^20^ Therefore, there is greater merit in identifying younger people at risk of diabetes or with undiagnosed diabetes.

While there are debates about the use of HbA1c in different ethnicities, important data from the Outcome Reduction with Initial Glargine Intervention (ORIGIN) trial of 12 527 people reported that the strong relationship between A1C and fasting plasma glucose (FPG) in people with moderate dysglycemia (5.6–9.0 mmol/L) is not significantly affected by ethnic or geographical differences.^21^ This range includes those with pre-diabetes, and as such the findings lend strong confidence that HbA1c levels in different ethnicities reflect similar dysregulation in glycemia levels.

In the UK, risk screening is supposed to link a risk score to identify those at highest risk, followed by formal glycemia testing either in fasted state (using fasting glucose) or any time of the day (HbA1c) in those with higher diabetes risk scores. Our work suggests that such risk scores may not be needed for middle-aged and old-aged South Asian or black individuals in whom the yield appears sufficiently high to consider single-stage mass screening using HbA1c. However, further studies are required to determine feasibility, uptake, and cost-effectiveness.

The work has some notable strengths, with the size and coverage of the study surpassing all prior studies in this area. Even so, we accept there are some limitations to our study. The sociodemographic representativeness of the UK Biobank is not identical to the general population.^22^ We addressed this partially by standardizing our estimates against the UK general population in terms of age, sex, and ethnic group distribution and we obtained very similar results. However, estimates of prevalence in the UK general population should still be taken with caution, as UK Biobank participants are less likely to be from a deprived area, less likely to be obese and to have a better lifestyle, and there is evidence of a ‘healthy volunteer’ selection bias.^22^ Indeed, it may be that the UK Biobank underestimates glycemia risks in some ethnicities. We had insufficient statistical power to include some ethnic groups, such as Arabs, and were obliged to amalgamate others. HbA1c values were also not available in a small proportion of people, but the level of missing data was comparable between ethnic groups and is unlikely to be systematic. We also recognize that HbA1c can sometimes be erroneous in people with certain hemoglobinopathies, but notably hemoglobin A1c was reportable in the presence of HbS, HbC, HbD, and HbE traits for the assay method used in the UK Biobank. We also recognize the a cut-off age of 40 years old means that younger people at risk of undiagnosed diabetes are not captured, but even so the results in other age groups should still be valid. Finally, the distribution of HbA1c was positively skewed and it is possible that those in the lower ranges of the HbA1c could be a dynamic group that may revert into ‘normal’ ranges.

We conclude that HbA1c is extremely efficient at identifying pre-diabetes and undiagnosed diabetes in middle-aged and old-aged South Asian and black individuals. Therefore, consideration should be given to single-stage mass screening of these high-risk populations using HbA1c.

## Data Availability

Data may be obtained from a third party and are not publicly available. UK Biobank data can be requested by bona fide researchers for approved projects, including replication, through https://www.ukbiobank.ac.uk/.
