# Supplementary material for: Ethnic differences in prevalence of actionable HbA1c levels in UK Biobank: implications for screening
Source: BMJ Open Diabetes Res Care. 2021 Aug 5;9(1):e002176. doi: 10.1136/bmjdrc-2021-002176 (PMC8344314; doi:10.1136/bmjdrc-2021-002176)
Supplement: Supplementary data [file bmjdrc-2021-002176supp001.pdf]

Ethnic differences in prevalence of actionable HbA1c levels in UK Biobank: implications for screening

Jana J Anderson, Paul Welsh, Frederick K Ho, Lyn D Ferguson, Claire E Welsh, Pierpaolo Pellicori, John GF Cleland, John Forbes, Stamatina Iliodromiti, James Boyle, Robert Lindsay, Carlos Celis-Morales, Stuart Robert Gray, Srinivasa Vittal Katikireddi, Jason Martin Regnald Gill, Jill P Pell, Naveed Sattar

SUPPLEMENTAL MATERIAL

Supplemental Table S1. Crude prevalence of pre- and undiagnosed diabetes and crude yield of HbA1c in UK Biobank by ethnic group excluding participants with cardiovascular disease.

	White N=397,354	South Asian N=6,937	Black N=5,355	Chinese N=1,337	Overall N=410,983
	N (%)	N (%)	N (%)	N (%)	N (%)
Pre-diabetes	11,807 (3.0)	712 (10.3)	713 (13.3)	103 (7.7)	13,335 (3.2)
Undiagnosed diabetes	2,532 (0.6)	239 (3.4)	154 (2.9)	15 (1.1)	2,940 (0.7)
Pre- or undiagnosed diabetes	14,339 (3.6)	951 (13.7)	867 (16.2)	118 (8.8)	16,275 (4.0)
	Yield	Yield	Yield	Yield	Yield
Pre-diabetes	1 in 34	1 in 10	1 in 8	1 in 13	1 in 31
Undiagnosed diabetes	1 in 157	1 in 29	1 in 35	1 in 89	1 in 140
Pre- or undiagnosed diabetes	1 in 28	1 in 7	1 in 6	1 in 11	1 in 25

N: number

Yield: Average number needing to be tested to identify one case

Anderson JJ, *et al. BMJ Open Diab Res Care* 2021; 9:e002176. doi: 10.1136/bmjdrc-2021-002176

Supplementary Table S2. Association between ethnic groups and pre- and undiagnosed diabetes

Prediabetes	Model 1<br>OR (95% CI)	P value	Model 2<br>OR (95% CI)	P value
White	1 Referent		1 Referent	
South Asian	3.63 (3.36-3.91)	<0.001	5.33 (4.90-5.80)	<0.001
Black	4.69 (4.34-5.07)	<0.001	5.43 (4.96-5.94)	<0.001
Chinese	2.47 (2.02-3.01)	<0.001	5.66 (4.58-6.99)	<0.001
Undiagnosed diabetes				
White	1 Referent		1 Referent	
South Asian	5.29 (4.65-6.02)	<0.001	7.05 (6.11-8.14)	<0.001
Black	4.43 (3.78-5.19)	<0.001	4.00 (3.37-4.76)	<0.001
Chinese	1.60 (0.98-2.66)	0.073	4.41 (2.63-7.39)	<0.001

OR Odds ratio; CI confidence interval

Model1: Unadjusted

Model 2: Adjusted for sociodemographic factors (sex, age, deprivation), BMI and lifestyle score, at baseline

Anderson JJ, *et al. BMJ Open Diab Res Care* 2021; 9:e002176. doi: 10.1136/bmjdrc-2021-002176
